# Activation of G protein-coupled estradiol receptor 1 in the dorsolateral striatum enhances motivation for cocaine and drug-induced reinstatement in female but not male rats

**DOI:** 10.1186/s13293-021-00389-w

**Published:** 2021-08-14

**Authors:** Jacqueline A. Quigley, Molly K. Logsdon, Brianna C. Graham, Kendra G. Beaudoin, Jill B. Becker

**Affiliations:** 1 Psychology Department, University of Michigan, Ann Arbor, MI 48109 USA; 2 Michigan Neuroscience Institute, University of Michigan, Ann Arbor, MI 48109 USA

**Keywords:** GPER1, Dorsolateral striatum, Sex differences, Motivation, Addiction, Cocaine

## Abstract

**Background:**

Estradiol potentiates drug-taking behaviors, including motivation to self-administer cocaine and reinstatement of drug-seeking after extinction in females, but not males. The dorsolateral stratum (DLS) is a region of the brain implicated in mediating drug-seeking behaviors and, more specifically, is a target brain area to study how estradiol regulates these behaviors. The estradiol receptors α, β, and G protein-coupled estradiol receptor 1 (GPER1) are all present in the DLS. In this study, the effects of activating GPER1 in the DLS on drug-seeking are investigated.

**Methods:**

Gonad-intact male and female rats were trained to self-administer cocaine (0.4 mg/kg/inf) on a fixed-ratio 1 schedule of reinforcement. For 4 weeks, animals underwent testing on a progressive ratio schedule of reinforcement to determine their motivation to attain cocaine. Halfway through progressive ratio testing, a selective agonist targeting GPER1 (G1) was administered intra-DLS to determine the contribution of GPER1 activation on motivation for cocaine. The effects of intra-DLS GPER1 activation on drug-induced reinstatement after extinction were subsequently determined.

**Results:**

Activation of GPER1, via intra-DLS G1 administration, potentiated females’ motivation to self-administer cocaine. There was no effect of prior G1 treatment on extinction of cocaine-taking in females; however, G1 treatment resulted in greater drug-induced reinstatement (10 mg/kg cocaine, i.p.). There were no effects of intra-DLS GPER1 activation observed on motivation for cocaine or cocaine-induced reinstatement of responding in males.

**Conclusions:**

These results support the conclusion that activation of GPER1 in the DLS enhances cocaine-seeking behaviors for female, but not male rats.

## Highlights

- Selective stimulation of the membrane estradiol receptor, GPER-1, in the dorsolateral striatum enhances motivation to self-administer cocaine in female rats.
- Selective stimulation of the membrane estradiol receptor, GPER-1, in the dorsolateral striatum enhances cocaine-induced reinstatement in female rats.
- GPER-1 stimulation in the dorsolateral striatum does not alter self-administration of cocaine or reinstatement of responding for cocaine in male rats.

## Introduction

The prevalence of adults who will develop a substance use disorder (SUD) is approximately 10%, although a much greater percentage of individuals will have exposure to elicit drug use at some point in their lifetime [19]. Many factors contribute to individual differences in escalation of drug-taking behavior and the propensity towards addiction. Biological sex is one component that affects individual differences in vulnerability to develop a SUD to psychostimulants, in particular [34]. For example, women escalate cocaine use more rapidly, report greater craving for cocaine, and have shorter cocaine-free periods compared to men [17, 45]. Women also have greater incidence of relapse, possibly due to stress-induced drug seeking that occurs more in women than men [3, 31].

There are sex differences in rodent models of addiction that are comparable to what is reported in the clinical literature (Jill B [7]). Female rats acquire cocaine self-administration more rapidly than males do, are more motivated to obtain cocaine, and take longer to extinguish cocaine-seeking behavior, compared to males ([23]; W J [28]; Wendy J [29, 37]). In females, but not males, the presence of estradiol potentiates sensitization to cocaine, acquisition and maintenance levels of drug intake, and reinstatement of cocaine-taking after extinction [16, 20, 30, 52]. Together, these data support that estradiol plays a role in increasing vulnerability to addiction-like behaviors in female rodents.

Recent evidence supports a modulatory role of estradiol on males’ preference for cocaine. Specifically, activation of the estradiol receptor subtype, G protein-coupled estradiol receptor 1 (GPER1), decreases conditioned place preference for cocaine and morphine in male rodents [33, 42]. As mentioned above, no studies thus far have determined an effect of estradiol treatment on males’ self-administration of cocaine, but this could be because prior studies have not investigated the contribution of individual estradiol receptor subtypes to drug self-administration in either sex.

Estradiol receptor subtypes including ERα, ERβ, and GPER1 are all expressed in the dorsal striatum of both males and females [2, 25, 33]. Given the recent evidence implicating GPER1 as an important neuronal target for mediating the rewarding properties of cocaine in males, this study was designed to determine whether GPER1 activation within the dorsolateral striatum (DLS) modulates motivation for cocaine in either sex. The current study used a progressive ratio self-administration paradigm to determine the contribution of GPER1 activation on motivation for cocaine and also evaluated the impact of DLS-GPER1 activation on drug-induced reinstatement in both female and male rats.

## Materials and methods

### Animals

A total of 25 male and 26 female gonad-intact Sprague-Dawley rats were used in this experiment. Animals were ordered from Charles River Breeding Laboratory (Portage, MI, USA) and were approximately 75 days old on arrival. Animals were maintained on a 14:10 light/dark cycle in a temperature-controlled climate of 72°F ± 2°F. Animals were housed individually in standard ventilated cages in the laboratory vivarium. In their home cages, rats had ad libitum access to water and phytoestrogen-free rat chow (2017 Teklad Global, 14% protein rodent maintenance diet, Harlan rat chow; Harlan Teklad, Madison, WI, USA). All animals were weighed daily to determine good health, and females were also vaginally lavaged daily to track estrous cycle. Estrous cycle was not interrupted by cocaine or G1 exposure. All animal care and experimental procedures were carried out in accordance with the National Institutes of Health guidelines on laboratory animal use and care, using a protocol approved by University of Michigan Institutional Use and Care of Animals Committee.

#### Stereotaxic surgery and treatment stylets

One week after arriving in the laboratory, rats underwent surgery for the implantation of bilateral guide cannula (purchased from P1 Technologies) aimed at the DLS (AP: +0.4 ML: +/−3.6 DV: −4.0). During surgery, 33-gauge solid stylets were inserted into the 26-gauge hollow guide cannula that were fixed on the animal’s heads. These stylets were flush with the bottom of the guide cannula and did not protrude further into the brain. Hollow treatment stylets were filled with solid vehicle or test drug and protruded from the guide cannula by 1mm to deliver treatment directly into the DLS. Control animals received 100% cholesterol (CHOL), and experimental animals received the selective GPER1 agonist G1, in cholesterol (10% G1:90% CHOL; dissolved in ethanol and evaporated to dryness prior to use). In order to insert stylets, rats were briefly anesthetized with 5% isoflurane. Post-mortem analyses confirmed correct placement of guide cannula into the DLS; no animals were excluded from analyses due to incorrect placement.

On the day of surgery, rats were injected with carprofen (5 mg/kg s.c) and 30 min later were anesthetized with ketamine (50 mg/kg i.p.) and dexmedetomidine (0.25 mg/kg i.p.). At the conclusion of the surgery, animals received atipamezole hydrochloride (0.5 mg/kg i.p.) and 3 ml 0.9% saline (s.c.). Every 24 h for 3 days post-surgery, animals were given carprofen (5 mg/kg s.c.) prophylactically for postoperative pain then monitored for an additional 7 days.

Stylets were prepared as previously described [5]. Pharmacological drugs were obtained from the following sources: Cholesterol (Santa Cruz Biotechnology, purity ≥ 92%) and G1 (Cayman Chemical, purity ≥ 98%). Previous studies report that G1 has no binding affinity for ERα or ERβ [1, 9].

#### Catheter surgery

One week after undergoing stereotaxic surgery, animals were fitted with indwelling jugular catheters that connected to a dorsal external port [13]. On the day of surgery, animals received carprofen (5 mg/kg s.c.) and 30 min later were anesthetized with 5% isoflurane in oxygen. Every 24 h for 3 days post-surgery, animals were given carprofen (5 mg/kg s.c.) prophylactically for postoperative pain. Animals were monitored for an additional 7 days before beginning self-administration behavioral testing.

Beginning 2 days after surgery and continuing everyday thereafter, catheters were flushed with 0.2 ml of gentamicin (3 mg/ml) and heparin (20 U/ml) to prevent infection and clotting, respectively. Prior to the beginning of each cocaine self-administration session, the catheters were also flushed with 0.1 ml of sterile saline. Once weekly, catheter patency was verified using 2.5 mg/kg methohexital sodium in sterile saline. Eight animals (4 males and 4 females) were removed from the experiment due to catheter failure. Two additional males and one female failed to continue after initial training (see below) when they failed the first catheter patency check).

#### Cocaine self-administration procedures chamber

**Fig. 1:**
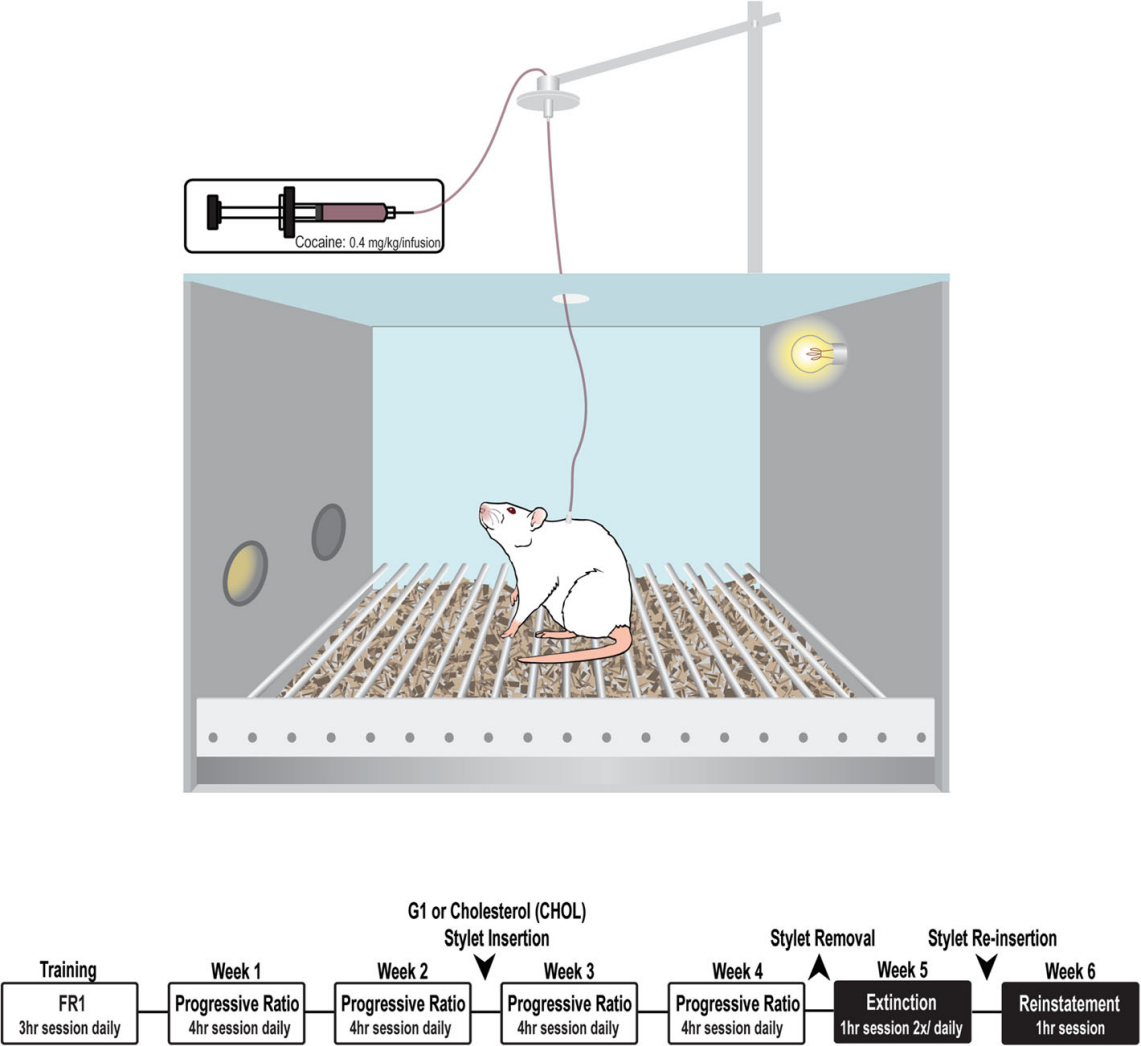
Illustration of self-administration operant chamber and timeline for self-administration training, progressive ratio, extinction, and reinstatement testing

Cocaine self-administration was performed in standard operant chambers (Med Associates, Inc., Georgia, VT, USA) for a maximum of 4 h per day, 5 days per week. As depicted in Fig. 1, each rat was able to move freely in the operant chamber, while connected to an infusion syringe via their dorsal catheter port. A house light turned on inside the chamber to signify the start of each self-administration session. Each chamber was also equipped with two nose poke ports. The active port had an illuminated light, while the other port had no light and was therefore “inactive.” A nose poke response in the active port resulted in an intravenous 50-μl infusion of 0.4 mg/kg/infusion cocaine HCl delivered over 2.8 s. There was no consequence of poking in the inactive port.

#### Training

Animals were tested 5 days a week with 2 days off each week. During week one, rats were trained to nose poke in the active port to self-administer cocaine on a fixed-ratio 1 schedule of reinforcement. Under this schedule, a response into the active port resulted in one infusion of cocaine followed by a 5-s timeout period of drug unavailability. If an animal nose poked during a timeout period, the nose poke was recorded but the animal did not receive an infusion of cocaine. Each training session was 3 h long or until an animal received a maximum of 15 infusions of cocaine. If an animal did not meet the 15-infusion threshold, they were given the remaining infusions one minute apart. By day 5 of training, all animals were earning 15 infusions of cocaine.

#### Progressive ratio

For four consecutive weeks thereafter, animals underwent a progressive ratio schedule of reinforcement that escalated through an exponential series: 1, 3, 6, 9, 12, 17, 24, 32, 42, 56, 73, 95, 124, 161, 208, … [35]. On this schedule, the number of nose pokes required increased exponentially and the consequence remained at a single cocaine infusion (0.4mg/kg/infusion). The final completed response ratio represents the animals’ breaking point. All progressive ratio tests lasted 4 h or until 1 h elapsed without the animal having earned the next infusion.

During weeks 3 and 4 of progressive ratio self-administration, animals received either G1 or CHOL intra-DLS (see Table 1 for treatment condition assignments) via their treatment stylets. Treatment conditions were assigned so that the average breaking point between each group did not differ for weeks 1 and 2 of progressive ratio testing. Treatment stylets were inserted after the final self-administration session of week 2 and remained through week 4, except for when they were briefly replaced with new stylets between weeks 3 and 4, in order to maintain a stable dose. Treatment stylets were removed at the conclusion of the last session of week 4.

**Table 1:**
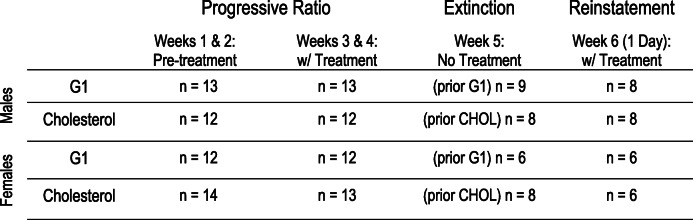
This table presents subjects per treatment condition at each stage of the self-administration paradigm

#### Extinction and reinstatement

During week 5, rats underwent 1-h extinction training twice per day for a total of 10 extinction training sessions in 5 days. Chamber conditions (i.e., house light and nose port light) were the same as during progressive ratio testing; however, rats did not receive an infusion of cocaine after nose poking. The rate of extinction was calculated as the difference between activate and inactive nose pokes per session.

New treatment stylets were introduced after the final extinction session. Treatment assignments were counterbalanced with prior G1 or CHOL exposure, to control for confounding effects of prior pharmacological manipulation. On day one of week 6, animals were tested for drug-induced reinstatement. At the start of the self-administration session, each animal received a 10 mg/kg i.p. injection of cocaine. Similar to during extinction, number of nose pokes were recorded; however, no consequence resulted from nose poking in either port.

#### Statistics

All statistical analyses were performed using GraphPad Prism v8.0 and IBM SPSS Statistics v27.0. Data were analyzed for general normality using the Shapiro-Wilk test but no corrections were needed. Muuchly’s Test was used to determine sphericity, and a Greenhouse-Geisser correction was used where sphericity was violated. Effect sizes for these tests are reported as Cohen’s d (d) and partial eta squared (n^2^p). The threshold for significance for all statistical tests was set to *p*<0.05.

**Fig. 2:**
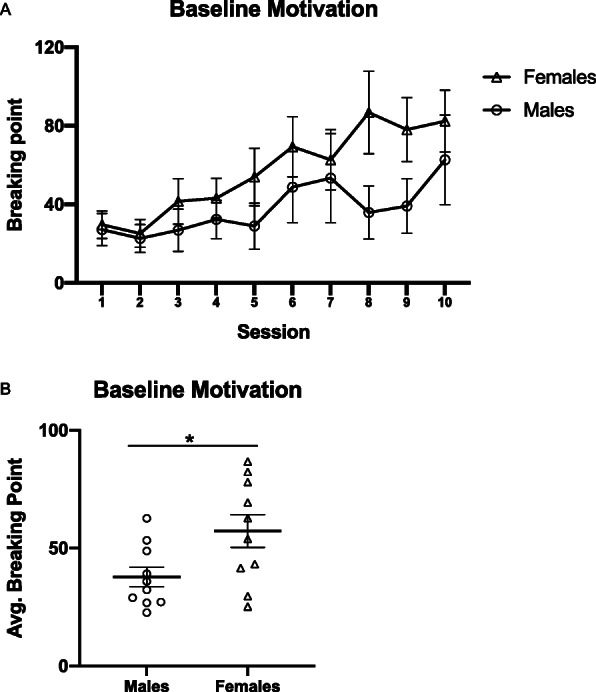
During weeks 1 and 2 of progressive ratio testing, **a** breaking point increases across self-administrations session for both sexes (*p* < 0.0001). **b** Females have a greater average breaking point across weeks compared to males (*p* = 0.0267). Data are presented as mean ± SEM

**Fig. 3:**
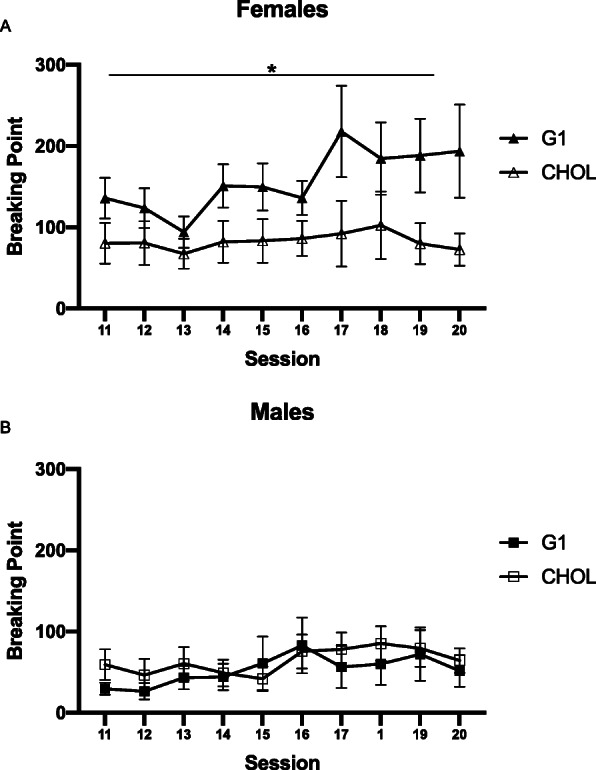
During weeks 3 and 4 of progressive ratio testing, G1 potentiates motivation for cocaine in **a** females (*p* = 0.0498) but not **b** males. Data are presented as mean ± SEM

**Fig. 4:**
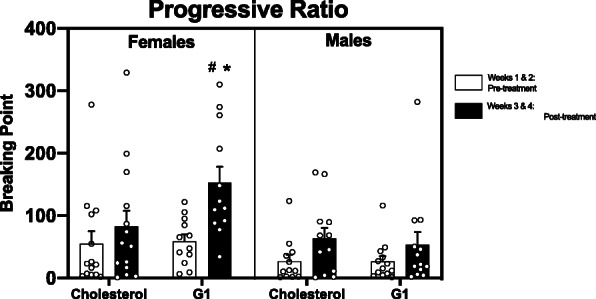
There are sex differences in the effects of GPER1 activation on motivation for cocaine. During weeks 3 and 4 of progressive ratio (PR), G1-treated females have significantly greater breaking point than they did during weeks 1 and 2, prior to treatment (*p* < 0.0001). G1-treated females also have a greater breaking point than G1-treated males, during weeks 3 and 4 of PR (*p* = 0.0039). Data are presented as mean ± SEM

**Fig. 5:**
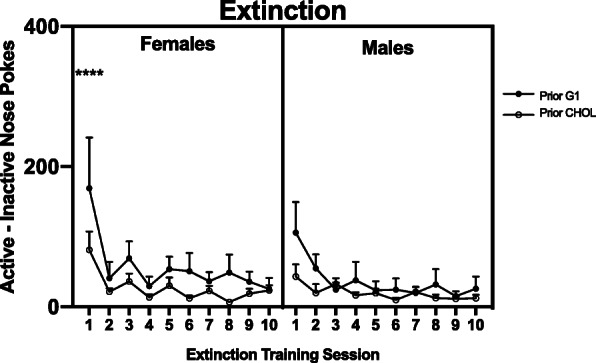
There is no effect of prior G1 treatment and no sex difference in the rates of cocaine self-administration. During the first extinction session only, prior G1-treated females are greater than all other groups (*p* < 0.0001). Data are presented as mean ± SEM

**Fig. 6:**
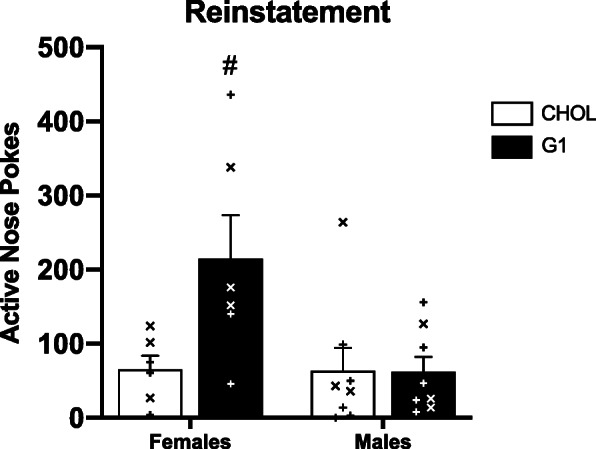
GPER1 activation enhances cocaine-induced reinstatement in females but not males. G1-treated females have a significantly greater number of active pokes than CHOL-treated females (*p* = 0.0460), G1 males (*p* = 0.0241), and CHOL males (*p* = 0.0259). Data are presented as mean ± SEM. Individual data points presented as “X” indicate prior G1 treatment and individual data point presented as “+” indicate prior CHOL treatment, during weeks 3 and 4 of self-administration

Sex differences in motivation were assessed across time, using a two-way repeated measures ANOVA (sex × session) and as average group differences, by using an unpaired *t* test (Fig. 2). Two-way repeated measures ANOVAs were also used to assess the effects of G1 versus CHOL on motivation within each sex (Fig. 3a, b). Three-way repeated measures ANOVAs were used to analyze sex differences in the effects of G1 versus CHOL on motivation (Fig. 4) and extinction (Fig. 5). A two-way ANOVA was used to analyze sex differences in the effects of G1 or CHOL on reinstatement (Fig. 6). In the case of a significant interaction, a Bonferroni multiple comparison test determined if there were significant group differences.

**Fig. 7:**
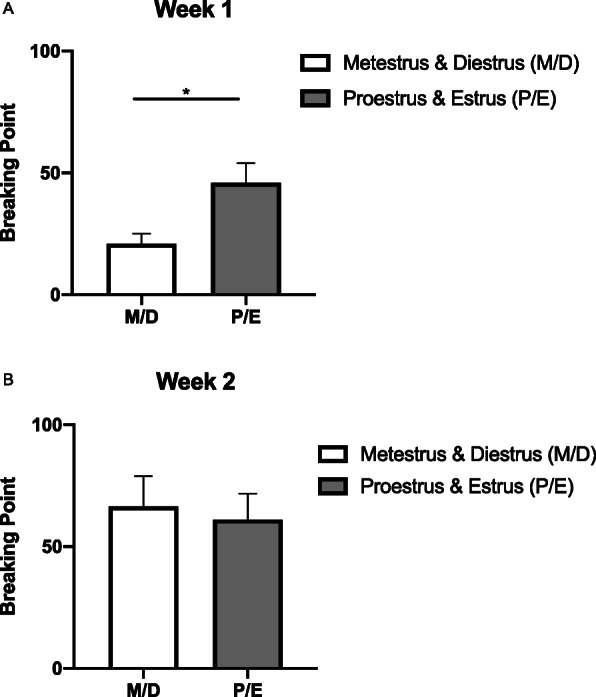
Females’ breaking point differs by phase of estrous cycle during **a** week 1 of progressive ratio self-administration (*p* = 0.0001) but not during **b** week 2. Data are presented as mean ± SEM

Finally, the effects of estrous cycle on motivation were analyzed by grouping non-estrus (metestrus and diestrus) versus estrus (proestrus and estrus) and comparing them using paired non-parametric Wilcoxon tests for week 1 and week 2 (Fig. 7).

## Results

During weeks 1 and 2 of progressive ratio testing, prior to any pharmacological manipulation, motivation for cocaine increased for both sexes (Fig. 2a). A two-way repeated measures ANOVA found a main effect of test session (F _(1.456,72.79)_ = 8.197; *p* = 0.0020; n^2^p = 0.141). Since previous studies have found sex differences on a progressive ratio schedule, an unpaired *t* test was performed to compare the mean breaking point values for males versus females across weeks 1 and 2 (t _(18)_ = 2.412; *p* = 0.0267; *d* = 1.078) (Fig. 2b). Thus, the average breaking point for females was greater than for males in the first 2 weeks of self-administration.

During weeks 3 and 4 of progressive ratio testing, DLS-GPER1 receptors were pharmacologically activated using G1 and motivation for cocaine was assessed within each sex (Fig. 3a, b). A two-way repeated measures ANOVA revealed a main effect of treatment for females (F _(1,24)_ = 4.267; *p* = 0.0498; n^2^p = 0.1509) but no main effect of day, and no treatment × day interaction. For males, there was no main effect of treatment or day, and no significant interaction.

As illustrated in Fig. 4, there are sex differences in the effects of G1 on breaking point for cocaine. A three-way repeated measures ANOVA revealed main effects of both sex (F _(1,47)_ = 6.973; *p* = 0.0112; n^2^p = 0.129) and timepoint (F _(1,47)_ = 33.14; *p* < 0.0001; n^2^p = 0.414). Additionally, there was a significant three-way interaction among sex × treatment condition × timepoint (F _(1,47)_ = 5.654; *p* = 0.0215; n^2^p = 0.107). Bonferroni multiple comparisons discovered significant group differences between G1-treated males and females’ post-treatment (*p* = 0.0039) as well as a significant difference in breaking point between timepoints in females treated with G1 (*p* < 0.0001).

As illustrated in Fig. 5, there was no effect of prior G1 exposure on rates of extinction. A three-way repeated measures ANOVA revealed a main effect of day (F _(9,243)_ = 5.840; *p* < 0.0001; n^2^p = 0.178) and a main effect of treatment condition (F _(1,27)_ = 4.317; *p* = 0.0474; n^2^p = 0.138). There were two significant interactions: sex × day (F _(9,243)_ = 2.563; *p* = 0.0078; n^2^p = 0.087) and sex × treatment condition (F _(9,243)_ = 2.982; *p* = 0.0022; n^2^p = 0.099). Bonferroni multiple comparisons indicated that the G1 females were significantly different from CHOL females (*p* < 0.0001) and both groups of males (*p* < 0.0001) on day 1 only. There were no group differences on any other day of extinction training between or within either sex.

Females treated with G1 also exhibited greater drug-induced reinstatement than did males (Fig. 6) regardless of prior treatment. A two-way ANOVA revealed a main effects of treatment condition (F _(1,24)_ = 5.189; *p* = 0.0319; n^2^p = 0.165) and sex (F _(1,24)_ = 4.745; *p* = 0.0394; n^2^p = 0.178). There was a significant sex × treatment condition interaction (F _(1,24)_ = 4.940; *p* = 0.0359; n^2^p = 0.171). Bonferroni multiple comparisons showed that G1-treated females were significantly different than CHOL females (*p* = 0.0460), G1 males (*p* = 0.0241), and CHOL males (*p* = 0.0259).

For females, phase of estrous cycle (metestrus/diestrus versus proestrus/estrus) had an effect on breaking point during week 1, but not during week 2 of progressive ratio (Fig. 7). For each female animal, mean breaking points during metestrus/diestrus days were compared to the mean breaking points during proestrus/estrus days. A paired *t* test was used to compare group means. During week 1, breaking point during proestrus/estrus was significantly greater than during metestrus/diestrus (*t*_(23)_ = 4.693; *p* < 0.0001; *d* = 0.782). There was no difference between estrous cycle timepoints during week 2 of progressive ratio (*t*_(24)_ = 0.8255; *p* = 0.172; *d* = 0.094).

## Discussion

We report here a sex difference in the effects of intra-DLS GPER1 activation on cocaine self-administration. For females, activation of GPER1 enhances females’ motivation for cocaine (i.e., breaking point), but this effect was not observed in males. Prior GPER1 activation did not alter females’ or males’ rates of extinction. However, females with intra-DLS GPER1 activation also show greater cocaine-induced reinstatement of drug-seeking behavior compared to control females. The effects of GPER1 activation on reinstatement in females were also not observed in males. Together, these findings indicate that estradiol may be enhancing vulnerability to addiction in females, at least in part, by acting on GPER1.

While this is the first study to show a role of GPER1 on cocaine self-administration specifically, previous research has found that estradiol enhances cocaine intake and motivation for cocaine (Jill B [6, 20, 32, 52]). Additionally, for female rodents, drug-associated cues acquire a higher incentive value when they are initially presented during estrus versus non-estrus [22]. Although the current study did not investigate the association of cue-learning, an effect of estrous cycle during initial stages of cocaine self-administration in females was found. During week 1 of progressive ratio testing, females show greater motivation to attain cocaine during proestrus/estrus compared to metestrus/diestrus. The lack of effect of estrous cycle in the succeeding weeks is likely due to the enhanced propensity to take cocaine overall.

We found that there were no differences in extinction rates between males and females or between prior treatment conditions beyond day 1 of extinction training. Prior studies have shown that estradiol is necessary for learning and extinction of cocaine-seeking in females [43]. Given that animals in the current study are gonad-intact and have circulating estradiol, it is not surprising that they extinguished at similar rates. It was important in the current study that animals extinguish similarly in order to compare rates of reinstatement.

Estradiol enhances females’ reinstatement of cocaine self-administration [16]. This effect had previously been shown to be regulated by ERβ, and not ERα, but this study was done via peripheral injections and did not investigate role of GPER1 on reinstatement [26]. Our study supports the idea that the DLS is a target region for estradiol’s effects on reinstatement in females.

Sex differences in drug-taking and cocaine reward are, in part, regulated by the interactions between estradiol and the dopamine system [10, 24, 51]. In vitro and in vivo studies have shown that estradiol can act directly on the dorsal striatum to enhance stimulated dopamine release and amphetamine-induced dopamine release in dorsal striatal tissue from female but not male rats [4, 11, 38, 39, 41, 46]. In vivo studies showed that systemic estradiol treatment in gonadectomized rats increases cocaine-induced dopamine levels in microdialysis of the dorsal striatum of ovariectomized females, but not castrated males [14]. Given the direct effect of intra-DLS GPER1 activation on cocaine-seeking in females seen in this study, we hypothesize that GPER1 could be, in part, modulating the effects of estradiol on the cocaine-induced increase in dopamine. Future studies should investigate this mechanism in both sexes.

In the current study, we did not see a protective effect of GPER1 activation on males’ motivation for cocaine, as both G1- and CHOL-treated males show increased motivation over time. However, we have previously reported that intra-DLS GPER1 activation attenuates cocaine conditioned place preference in males [33]. Previous research that demonstrated that the DLS is necessary for stimulus-response learning in males, along with the current results, suggest that the timing of pharmacological activation of intra-DLS GPER1−, relative to initial drug exposure, is important for GPER1’s effects on motivation for cocaine [47, 48]. In our earlier study, GPER1 receptors in the DLS were activated or inhibited prior to the initial cocaine treatment, whereas in the current study, animals begin taking cocaine 3 weeks prior to administration of the GPER1 agonist. Additional studies are needed to determine whether activating GPER1 receptors intra-DLS before rats are trained to self-administer cocaine would affect the subsequent motivation and propensity to self-administer in males and/or females.

As discussed above, in our prior study, we reported that intra-DLS GPER1 attenuated males’ preference for, or “liking,” of cocaine. In our previous study, we also showed that the same dose of intra-DLS G1 used in the study here attenuated preference for saccharine in males, but not females, indicating a general effect of G1 in males to decrease preference [33]. In this study, we have shown that there is no effect of intra-DLS GPER1 on “wanting” cocaine in males. The neurobiological mechanisms of “liking” a drug are discrete from “wanting;” that is, one may not necessarily like a drug but still crave and consume it. These dissociable mechanisms and are mediated by opioidergic and dopaminergic signaling, respectively [8, 36]. We speculate that the interactions of GPER1 on opioid and dopamine signaling are different for females and males, and this could be contributing to sex-dependent behavioral outcomes related to propensity to addiction.

There is circumstantial evidence for sex differences in the circuitry for “wanting” and “liking.” In females, estradiol acts on GABAergic neurons to disinhibit dopaminergic neurons and increase dopamine levels in the striatum [49]. This enhanced neurotransmission of dopamine is presumably responsible for females’ more rapid escalation of self-administration and enhanced motivation to attain psychostimulants [14, 50]. Directly below the dorsal striatum is the nucleus accumbens shell which is an opioid hedonic hotspot that regulates “liking” [12]. In males, pharmacological studies have implicated mu-opioid receptor functioning in the shell subregion to regulate responses for palatable food and cocaine [40, 44]. The direct interactions of GPER1 on μ-opioid receptor function in the dorsal and ventral striatum are yet to be investigated. However, there is some evidence for crosstalk between these receptors including GPER1 activation rapidly downregulating μ-opioid receptors in the arcuate nucleus as well as eliciting phosphorylation of μ-opioid receptors in human neuroblastoma SH-SY5Y cells [15, 27].

It is also possible that the sex differences in the effect of G1 on preference for cocaine vs. motivation for cocaine are due to different dose-response sensitivities of males and females, reflecting differences in cellular or subcellular distribution. One limitation of this study is only a single concentration of G1 was used, and only a single dose of cocaine was used during self-administration and cocaine-induced reinstatement. GPER1 has been shown to have a bell-shaped dose-response curve for social recognition in mice [18]. It will be important to test other doses in males in case this concentration of G1 was too high or too low to see an effect on motivation.

In summary, the present study confirmed previous findings that there are sex differences related to motivation to attain drugs of abuse. As discussed above, a large body of work has supported that estradiol enhances females’ vulnerability towards addiction but has not necessarily unveiled which estradiol receptor subtypes are responsible for the behavioral effects seen in females. The results of this study support a novel role of GPER1 in females and provide a future target for preclinical research as well as clinical research targeted at therapeutics for addiction.

## Perspectives and significance

It is vital that we better understand the neurobiological mechanisms contributing to drug addiction and relapse in women, given that they are more sensitive to environmental cues and more susceptible to spontaneous relapse [21, 34]. Increased drug-seeking induced by estradiol in females has been well established, and the current study aids to this body of knowledge by identifying a role for GPER1, specifically. In this study, activation of GPER1 in the DLS not only enhances motivation for cocaine in females, but also increases drug-induced reinstatement. The information gained here may be used to target treatment for addiction via selective estradiol receptor modulators.

## Data Availability

Please contact the first or last author for data or materials requests.

## Abbreviations

SUD: Substance use disorder
GPER1: G protein-coupled estradiol receptor 1
DLS: Dorsolateral striatum
s.c.: Subcutaneous
i.p.: Intraperitoneal
CHOL: Cholesterol

## References

[1] Albanito L, Madeo A, Lappano R, Vivacqua A, Rago V, Carpino A, Oprea TI, Prossnitz ER, Musti AM, Andò S, Maggiolini M (2007). G protein-coupled receptor 30 (GPR30) mediates gene expression changes and growth response to 17beta-estradiol and selective GPR30 ligand G-1 in ovarian cancer cells. Cancer Res.

[2] Almey A, Milner TA, Brake WG (2016). Estrogen receptor α and G-protein coupled estrogen receptor 1 are localized to GABAergic neurons in the dorsal striatum. Neurosci Lett.

[3] Back SE, Brady KT, Jackson JL, Salstrom S, Zinzow H (2005). Gender differences in stress reactivity among cocaine-dependent individuals. Psychopharmacology.

[4] Becker JB (1990). Direct effect of 17 beta-estradiol on striatum: sex differences in dopamine release. Synapse.

[5] Becker JB, Snyder PJ, Miller MM, Westgate SA, Jenuwine MJ (1987). The influence of estrous cycle and intrastriatal estradiol on sensorimotor performance in the female rat. Pharmacol Biochem Behav.

[6] Becker JB, Hu M (2008). Sex differences in drug abuse. Front Neuroendocrinol.

[7] Becker JB, Koob GF (2016). Sex differences in animal models: focus on addiction. Pharmacol Rev.

[8] Berridge KC (2007). The debate over dopamine’s role in reward: the case for incentive salience. Psychopharmacology.

[9] Bologa CG, Revankar CM, Young SM, Edwards BS, Arterburn JB, Kiselyov AS, Parker MA, Tkachenko SE, Savchuck NP, Sklar LA, Oprea TI, Prossnitz ER (2006). Virtual and biomolecular screening converge on a selective agonist for GPR30. Nat Chem Biol.

[10] Calipari ES, Juarez B, Morel C, Walker DM, Cahill ME, Ribeiro E, Roman-Ortiz C, Ramakrishnan C, Deisseroth K, Han MH, Nestler EJ (2017). Dopaminergic dynamics underlying sex-specific cocaine reward. Nat Commun.

[11] Castner SA, Xiao L, Becker JB (1993). Sex differences in striatal dopamine: in vivo microdialysis and behavioral studies. Brain Res.

[12] Castro DC, Berridge KC (2014). Opioid hedonic hotspot in nucleus accumbens shell: mu, delta, and kappa maps for enhancement of sweetness “liking” and “wanting”. J Neurosci.

[13] Cummings JA, Gowl BA, Westenbroek C, Clinton SM, Akil H, Becker JB (2011). Effects of a selectively bred novelty-seeking phenotype on the motivation to take cocaine in male and female rats. Biol Sex Diff.

[14] Cummings JA, Jagannathan L, Jackson LR, Becker JB (2014). Sex differences in the effects of estradiol in the nucleus accumbens and striatum on the response to cocaine: neurochemistry and behavior. Drug Alcohol Depend.

[15] Ding X, Gao T, Gao P, Meng Y, Zheng Y, Dong L, Luo P, Zhang G, Shi X, Rong W (2019). Activation of the G protein-coupled estrogen receptor elicits store calcium release and phosphorylation of the Mu-opioid receptors in the human neuroblastoma SH-SY5Y cells. Front Neurosci.

[16] Doncheck EM, Urbanik LA, DeBaker MC, Barron LM, Liddiard GT, Tuscher JJ, Frick KM, Hillard CJ, Mantsch JR (2018). 17β-Estradiol potentiates the reinstatement of cocaine seeking in female rats: role of the prelimbic prefrontal cortex and cannabinoid type-1 receptors. Neuropsychopharmacology.

[17] Elman I, Karlsgodt KH, Gastfriend DR (2001). Gender differences in cocaine craving among non-treatment-seeking individuals with cocaine dependence. Am J Drug Alcohol Abuse.

[18] Gabor C, Lymer J, Phan A, Choleris E (2015). Rapid effects of the G-protein coupled oestrogen receptor (GPER) on learning and dorsal hippocampus dendritic spines in female mice. Physiol Behav.

[19] Grant BF, Saha TD, Ruan WJ, Goldstein RB, Chou SP, Jung J, Zhang H, Smith SM, Pickering RP, Huang B, Hasin DS (2016). Epidemiology of DSM-5 drug use disorder: results from the National Epidemiologic Survey on Alcohol and Related Conditions-III. JAMA Psychiatry.

[20] Jackson LR, Robinson TE, Becker JB (2006). Sex differences and hormonal influences on acquisition of cocaine self-administration in rats. Neuropsychopharmacology.

[21] Janes AC, Pizzagalli DA, Richardt S, de Frederick B, Holmes AJ, Sousa J (2010). Neural substrates of attentional bias for smoking-related cues: an FMRI study. Neuropsychopharmacology.

[22] Johnson AR, Thibeault KC, Lopez AJ, Peck EG, Sands LP, Sanders CM, Kutlu MG, Calipari ES (2019). Cues play a critical role in estrous cycle-dependent enhancement of cocaine reinforcement. Neuropsychopharmacology.

[23] Kippin TE, Fuchs RA, Mehta RH, Case JM, Parker MP, Bimonte-Nelson HA, See RE (2005). Potentiation of cocaine-primed reinstatement of drug seeking in female rats during estrus. Psychopharmacology.

[24] Kokane SS, Perrotti LI (2020). Sex differences and the role of estradiol in mesolimbic reward circuits and vulnerability to cocaine and opiate addiction. Front Behav Neurosci.

[25] Krentzel AA, Willett JA, Johnson AG, Meitzen J (2021). Estrogen receptor alpha, G-protein coupled estrogen receptor 1, and aromatase: developmental, sex, and region-specific differences across the rat caudate-putamen, nucleus accumbens core and shell. J Comp Neurol.

[26] Larson EB, Carroll ME (2007). Estrogen receptor beta, but not alpha, mediates estrogen’s effect on cocaine-induced reinstatement of extinguished cocaine-seeking behavior in ovariectomized female rats. Neuropsychopharmacology.

[27] Long N, Serey C, Sinchak K (2014). 17β-estradiol rapidly facilitates lordosis through G protein-coupled estrogen receptor 1 (GPER) via deactivation of medial preoptic nucleus μ-opioid receptors in estradiol primed female rats. Hormones Behav.

[28] Lynch WJ, Carroll ME (1999). Sex differences in the acquisition of intravenously self-administered cocaine and heroin in rats. Psychopharmacology.

[29] Lynch WJ (2008). Acquisition and maintenance of cocaine self-administration in adolescent rats: effects of sex and gonadal hormones. Psychopharmacology.

[30] Martinez LA, Gross KS, Himmler BT, Emmitt NL, Peterson BM, Zlebnik NE, et al. Estradiol facilitation of cocaine self-administration in female rats requires activation of mGluR5. ENeuro. 2016;3(5). 10.1523/ENEURO.0140-16.2016. 10.1523/ENEURO.0140-16.2016 PMC5079229 27822496

[31] McKay JR, Rutherford MJ, Cacciola JS, Kabasakalian-McKay R, Alterman AI (1996). Gender differences in the relapse experiences of cocaine patients. J Nervous Mental Dis.

[32] Perry AN, Westenbroek C, Becker JB (2013). Impact of pubertal and adult estradiol treatments on cocaine self-administration. Hormones Behav.

[33] Quigley JA, Becker JB (2021). Activation of G-protein coupled estradiol receptor 1 in the dorsolateral striatum attenuates preference for cocaine and saccharin in male but not female rats. Hormones Behav.

[34] Quigley JA, Logsdon MK, Turner CA, Gonzalez I, Leonardo N, Becker JB (2021). Sex differences in vulnerability to addiction. Neuropharmacology.

[35] Richardson NR, Roberts DC (1996). Progressive ratio schedules in drug self-administration studies in rats: a method to evaluate reinforcing efficacy. J Neurosci Methods.

[36] Robinson TE, Berridge KC (1993). The neural basis of drug craving: an incentive-sensitization theory of addiction. Brain Res Rev.

[37] Roth ME, Carroll ME (2004). Sex differences in the escalation of intravenous cocaine intake following long- or short-access to cocaine self-administration. Pharmacol Biochem Behav.

[38] Shams WM, Cossette M-P, Shizgal P, Brake WG (2018). 17β-estradiol locally increases phasic dopamine release in the dorsal striatum. Neurosci Lett.

[39] Shams WM, Sanio C, Quinlan MG, Brake WG (2016). 17β-Estradiol infusions into the dorsal striatum rapidly increase dorsal striatal dopamine release in vivo. Neuroscience.

[40] Simmons D, Self DW (2009). Role of mu- and delta-opioid receptors in the nucleus accumbens in cocaine-seeking behavior. Neuropsychopharmacology.

[41] Song Z, Yang H, Peckham EM, Becker JB. Estradiol-induced potentiation of dopamine release in dorsal striatum following amphetamine administration requires estradiol receptors and mGlu5. ENeuro. 2019;6(1). 10.1523/ENEURO.0446-18.2019. 10.1523/ENEURO.0446-18.2019 PMC6374122 30766916

[42] Sun K, Wang F, Ma L, Ren X, Zhang C, Rong W, Sun T (2020). Genetic knockout of the G protein-coupled estrogen receptor 1 facilitates the acquisition of morphine-induced conditioned place preference and aversion in mice. Biochem Biophys Res Commun.

[43] Twining RC, Tuscher JJ, Doncheck EM, Frick KM, Mueller D (2013). 17β-estradiol is necessary for extinction of cocaine seeking in female rats. Learning & Memory.

[44] Ward HG, Nicklous DM, Aloyo VJ, Simansky KJ (2006). Mu-opioid receptor cellular function in the nucleus accumbens is essential for hedonically driven eating. Eur J Neurosci.

[45] Westermeyer J, Kopka S, Nugent S (1997). Course and severity of substance abuse among patients with comorbid major depression. Am J Addict.

[46] Xiao L, Jackson LR, Becker JB (2003). The effect of estradiol in the striatum is blocked by ICI 182,780 but not tamoxifen: pharmacological and behavioral evidence. Neuroendocrinology.

[47] Yin HH, Knowlton BJ, Balleine BW (2005). Blockade of NMDA receptors in the dorsomedial striatum prevents action-outcome learning in instrumental conditioning. Eur J Neurosci.

[48] Yin HH, Knowlton BJ, Balleine BW (2006). Inactivation of dorsolateral striatum enhances sensitivity to changes in the action-outcome contingency in instrumental conditioning. Behav Brain Res.

[49] Yoest KE, Cummings JA, Becker JB (2014). Estradiol, dopamine and motivation. Cent Nerv Syst Agents Med Chem.

[50] Yoest KE, Cummings JA, Becker JB (2019). Ovarian hormones mediate changes in adaptive choice and motivation in female rats. Front Behav Neurosci.

[51] Yoest KE, Quigley JA, Becker JB (2018). Rapid effects of ovarian hormones in dorsal striatum and nucleus accumbens. Hormones Behav.

[52] Zhao W, Becker JB (2010). Sensitization enhances acquisition of cocaine self-administration in female rats: estradiol further enhances cocaine intake after acquisition. Hormones Behav.

